# Meta-Analysis of Microsomal Epoxide Hydrolase Gene Polymorphism and Risk of Hepatocellular Carcinoma

**DOI:** 10.1371/journal.pone.0057064

**Published:** 2013-02-25

**Authors:** Jian-Hong Zhong, Bang-De Xiang, Liang Ma, Xue-Mei You, Le-Qun Li, Gui-Sheng Xie

**Affiliations:** 1 Hepatobiliary Surgery Department, Tumor Hospital of Guangxi Medical University, Nanning, People’s Republic of China; 2 General Surgery Department, The Third Affiliated Hospital of Guangxi Medical University, Nanning, People’s Republic of China; University of Modena & Reggio Emilia, Italy

## Abstract

**Background:**

Hepatocarcinogenesis is a complex process that may be influenced by many factors, including polymorphism in microsomal epoxide hydrolase (mEH). Previous work suggests an association between the Tyr113His and His139Arg mEH polymorphisms and susceptibility to hepatocellular carcinoma (HCC), but the results have been inconsistent.

**Methods:**

PubMed, EMBASE, Google Scholar and the Chinese National Knowledge Infrastructure databases were systematically searched to identify relevant studies. A meta-analysis was performed to examine the association between Tyr113His and His139Arg mEH polymorphism and susceptibility to HCC. Odds ratios (ORs) and 95% confidence intervals (95% CIs) were calculated.

**Results:**

Eleven studies were included in the meta-analysis, involving 1,696 HCC cases and 3,600 controls. The 113His- mEH allele was significantly associated with increased risk of HCC based on allelic contrast (OR = 1.35, 95% CI = 1.04–1.75, p = 0.02), homozygote comparison (OR = 1.65, 95% CI = 1.07–2.54, p = 0.02) and a recessive genetic model (OR = 1.54, 95% CI = 1.21–1.96, p<0.001), while individuals carrying the Arg139Arg mEH genotype had no association with increased or decreased risk of HCC.

**Conclusion:**

The 113His- allele polymorphism in mEH may be a risk factor for hepatocarcinogenesis, while the mEH 139Arg- allele may not be a risk or protective factor. There is substantial evidence that mEH polymorphisms interact synergistically with other genes and the environment to modulate risk of HCC. Further large and well-designed studies are needed to confirm these conclusions.

## Introduction

As the most frequent primary cancer of the liver, hepatocellular carcinoma (HCC) is a significant cause of cancer morbidity and mortality worldwide, and treatment options are limited. The estimated incidence of new HCC cases each year is more than 0.5 million [1]. Sub-Saharan Africa and Southeast Asia are the regions with highest incidence of HCC (>20 per 100,000 people) [2]. Epidemiologically, HCC is strongly associated with hepatitis B virus (HBV) and hepatitis C virus (HCV) infection, alcohol consumption, and aflatoxin B1 (AFB) contamination of foodstuffs, among other factors [3]–[4]. In developing countries, AFB together with chronic HBV infection contributes to the high incidence of HCC. However, not all individuals with these factors appear to have the same risk of developing HCC. HCC exhibits a high degree of genetic heterogeneity: multiple molecular pathways may give rise to subsets of hepatocellular neoplasms.

HCC pathogenesis remains incompletely understood. It is known to involve chronic inflammation, hepatocyte hyperplasia and ultimately malignant transformation [5]. Current thinking is that HCC is a multifactorial disease, the etiology of which involves various host and environmental factors. Moreover, host and environmental factors may interact synergistically in HCC pathogenesis and progression.

The liver has a complex detoxification system. In this complex system, several enzymes participate in the metabolism of both exogenous and endogenous metabolites produced by oxidative stress in chronic infections, such as HBV and HCV infections. The genes encoding these enzymes are polymorphic, and the variant sequences lead to enzymes with altered activity. Any genetic defect in these xenobiotic-metabolizing enzymes may lead to accumulation of viral integration products and xenobiotics. This should increase the risk of liver diseases, such as cirrhosis and HCC.

Microsomal epoxide hydrolase (mEH) is one of these enzymes. As a phase II metabolic enzyme, mEH is involved in the hydrolysis of various epoxides and deleterious chemical epoxide intermediates generated by phase I oxidation reactions [6]–[7]. Since epoxides are highly reactive oxidative metabolites, mEH is considered to act on the most toxicologically active forms of drugs and environmental chemicals. Despite its important protective function, its net effects on the body can be complex, since it plays a dual role of procarcinogen detoxifier and activator [8].

The gene encoding mEH consists of nine exons and eight introns. Two relatively frequent single nucleotide polymorphisms occur in the coding region: Tyr113His in exon 3 and His139Arg in exon 4. These polymorphisms modify protein stability: Tyr113His decreases enzyme activity, while His139Arg increases it [9].

In 1995, Mcglynn and coworkers [10] explored the association between mEH polymorphism and risk of HCC. They found that the Tyr113His mEH gene polymorphism is associated with risk of developing HCC. The study also found a synergistic increase in the risk of HCC when HBV infection and susceptible genotype occurred together [10]. More recently, epidemiological studies have evaluated the association between the Tyr113His and His139Arg mEH gene polymorphisms and risk of HCC in diverse ethnicities, with inconsistent results [11]–[20]. Some of these studies also revealed synergistic gene-gene [13], [17]–[19] and gene-environment [10], [16], [20] interactions. A single case-control study may fail to completely demonstrate these complicated genetic relationships because of small sample size. In order to provide stronger evidence of the effects of these two mEH polymorphisms on HCC risk, we carried out a meta-analysis by combining data from numerous published studies.

## Methods

### Search Strategy

All case-control studies of mEH polymorphism and HCC risk published up to August 31, 2012 were identified through systematic searches in PubMed, EMBASE, Google Scholar and the Chinese National Knowledge Infrastructure (CNKI) databases using English and Chinese. The search terms used were: *mEH; HYL1; EPHX; microsomal epoxide hydrolase;* these four terms in combination with *polymorphism, variation, genotype, genetic* and *mutation;* and all of the above terms in combination with *hepatocellular carcinoma, HCC, liver cancer, liver tumor, liver neoplasms* and *hepatic tumor*. The references of each article identified were also manually searched to identify additional relevant publications.

### Inclusion Criteria

A study was included in the meta-analysis if it satisfied the following criteria: (a) it assessed the association between HCC and the Tyr113His and His139Arg mEH gene polymorphisms, (b) it used a case-control design, and (c) it provided sufficient published data for estimating an odds ratio (OR) with a 95% confidence interval (95% CI). In the case of multiple studies based on the same population, we selected the study with the largest number of participants.

### Data Extraction

Literature searches and identification of eligible articles based on the inclusion criteria were carried out independently by two authors (JHZ and BDX). These authors independently extracted the following data: first author’s name, year of publication, ethnicity or country, source of controls (hospital- or population-based), exon number, numbers and genotypes of cases and controls, and Hardy-Weinberg equilibrium (HWE) of controls. Discrepancies were resolved by consensus.

### Statistical Methods

The unadjusted OR with 95% CI was used to assess the strength of the association between the Tyr113His and His139Arg mEH polymorphisms and HCC risk based on the genotype frequencies in cases and controls. Subgroup analysis stratified by ethnicity was performed. Ethnicity was categorized as Chinese and mixed. The meta-analysis examined the association of different genotypes at Tyr113His mEH with HCC risk by comparing the 113 Tyr allele (113T-allele) with the 113 His allele (113H-allele), the homozygous comparison (His113His vs. Tyr113Tyr), and recessive and dominant genetic model. The same approach was used to examine the association of His139Arg mEH and HCC risk.

All statistical tests for this meta-analysis were performed using RevMan 5.0 and Stata 11.0 softwares. Fixed-effect and random-effect models were used to calculate a pooled OR. The statistical significance of the pooled OR was determined using the Z-test, and P<0.05 was considered statistically significant. The assumption of heterogeneity was evaluated by applying a chi square-based Q-test among the studies. P>0.10 for the Q-test indicates a lack of statistical heterogeneity, suggesting the variability in effect sizes is larger than that expected from chance alone [21]. In these cases, a pooled OR was calculated for each study using the fixed-effects model. Otherwise, the random-effect model was used. To assess the reliability of the outcomes in the meta-analysis, a sensitivity analysis was performed by excluding one study at a time. Small-study bias was assessed by Harbord’s modified test [22]. HWE in the control group was assessed using the asymptotic test, with P<0.05 considered significant.

## Results

### Description of Studies

**Figure 1:**
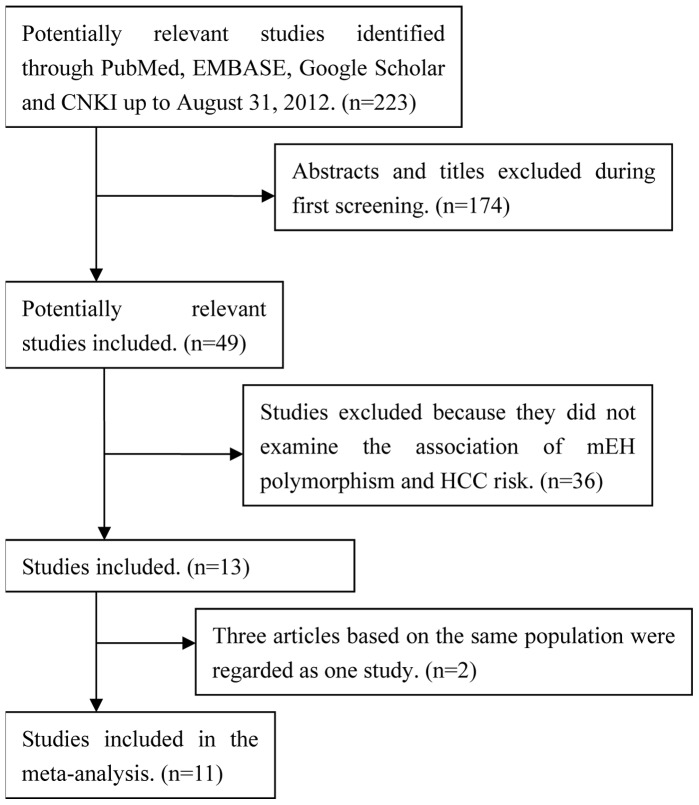
Flow chart of study selection. CNKI, Chinese National Knowledge Infrastructure; mEH, microsomal epoxide hydrolase; HCC, hepatocellular carcinoma.

A total of 223 potentially relevant publications up to August 31, 2012 were systematically identified through PubMed, EMBASE, Google Scholar and CNKI. After screening the title and/or abstracts, 174 (78%) were excluded because they did not satisfy the inclusion criteria. An additional 36 publications were excluded because they did not examine Tyr113His and His139Arg mEH polymorphisms or they were review articles. Three publications [19], [23], [24] were based on the same participants with HCC, so they were considered as one study. In the end, 11 studies [10]–[20] were included in this meta-analysis based on our search strategy and inclusion criteria (Fig. 1).

We established a database according to the information extracted from each article. Detailed characteristics of the 11 studies are listed in Tables 1 and 2. Overall, 1,696 HCC cases and 3,600 controls were retrieved. Six of the studies involved Chinese subjects [10]–[11], [15]–[16], [18], [20]. Of these, three studies involving 232 cases and 322 controls were published in Chinese, while the other three involving 540 cases and 1021 controls were published in English. The populations of the remaining five studies came from the UK [12], Sudan [13], Italy [14], Gambia [17], and India [19]. All studies had a case-control design. Three studies [12], [14], [19] included a control population with liver disease (hospital-based control). These three studies [12], [14], [19] involved 202 HCC cases and 536 controls. The number of population-based controls was 3,064. However, some of the population-based controls (health population) had liver disease. Therefore, subgroup analysis by source of controls was not conducted. Of the total number of 5,296 subjects considered in the meta-analysis, at least 2,000 (37.8%) had one or more of the following: alcoholic liver disease, HBV or HCV infection, and cirrhosis. Most studies did not report detailed data on the percentages of their subjects with these background conditions. Therefore we could not define subgroups for meta-analysis based on liver disease, HBV/HCV infection or cirrhosis status.

**Table 1:** Main characteristics of studies about mEH exon 3 polymorphism included in the meta-analysis.

Study	Country	Source ofcontrol	P_HWE_	P_Frequency of_ _113His-allele_	Cases/Controls	No. of cases			No. of controls		
						risk	heterozygous	non-risk	risk	heterozygous	non-risk
Mcglynn 1995^10^	Chineseand Ghana	Healthy§	<0.001	<0.001	52/165	29	18	5	44	57	64
Shen 1997^11^	Chinese	Healthy§	0.001	0.03	39/67	21	13	4	27	20	20
Wong 2000^12^	UK	Healthyand ALD	0.087	0.42	46/264	5	23	18	20	127	117
Tiemersma 2001^13^	Sudan	Healthy§	0.007	0.21	112/194	14	28	68	15	50	128
Sonzogni 2002^14^	Italy	Healthy, HC,CH, andCirrhosis	0.0258	<0.001	93/400	26	38	29	57	159	184
McGlynn 2003^15^	Chinese	Healthy§	<0.001	0.86	231/256	118	49	64	113	87	56
Cao 2004^16^	Chinese	Healthy§	0.120	0.01	88/104	35	35	18	27	44	33
Kirk 2005^17^	Gambia	Healthy§	0.001	0.49	195/351	11	46	138	18	77	256
Long 2006^18^	Chinese	Healthy§	<0.001	<0.001	257/649	91	70	96	168	100	381
Kiran 2008^19^	Indian	Healthyand CH	0.280	0.005	63/343	12	25	26	100	161	82
He 2008^20^	Chinese	Healthy§	0.001	0.97	105/151	53	23	29	67	52	32

Note: ALD, alcoholic liver disease; CH, Chronic hepatitis; HC, hepatitis carrier; HWE, Hardy-Weinberg equilibrium; §, some were carriers of hepatitis B and/or C virus.

**Table 2:** Main characteristics of studies about mEH exon 4 polymorphism included in the meta-analysis.

Study	Country	Source ofcontrol	P_HWE_	P_Frequency of_ _139Arg-allele_	Cases/Controls	No. of cases			No. of controls		
						risk	heterozygous	non-risk	risk	heterozygous	non-risk
Wong 2000^12^	UK	Healthyand ALD	0.831	0.91	39/264	1	12	26	7	78	179
Tiemersma 2001^13^	Sudan	Healthy§	0.847	0.54	112/194	3	44	63	13	69	102
Sonzogni 2002^14^	Italy	Healthy, HC, CH,and Cirrhosis	0.876	0.22	93/400	4	22	67	15	131	254
McGlynn 2003^15^	Chinese	Healthy§	0.074	<0.001	231/256	3	31	197	3	79	174
Cao 2004^16^	Chinese	Healthy§	0.296	0.02	88/104	1	12	75	1	32	71
Kiran 2008^19^	Indian	Healthyand CH	0.221	0.37	63/343	16	31	16	64	155	124

Note: ALD, alcoholic liver disease; CH, Chronic hepatitis; HC, hepatitis carrier; HWE, Hardy-Weinberg equilibrium; §, some were carriers of hepatitis B and/or C virus.

All 11 studies described the Tyr113His polymorphism; however, only 6 studies [12]–[16], [19] described the His139Arg polymorphism. The distribution of genotypes among controls did not show HWE in several studies [10]–[11], [13]–[15], [17]–[18], [20] (Tables 1 and 2).

### Test of Heterogeneity

Table 3 shows the relationship between the Tyr113His mEH polymorphism and HCC risk. The statistical heterogeneity of Tyr113His mEH allelic contrast, homozygote comparison, and dominant and recessive genetic models was analyzed for all 11 studies. Statistically significant heterogeneity was observed in all studies except for the recessive genetic model for the Chinese subgroup. Therefore, random-effect models were used to analyze the OR.

**Table 3:** Overall and stratified meta-analyses of the association between mEH polymorphism Tyr113His and risk of hepatocellular carcinoma.

Genotype comparison	OR [95% CI]	Z (P value)	Heterogeneity of study design			Analysis model
			?^2^	df (P value)	I^2^	
**Total (1696 cases, 3600 controls)**						
113His-allele vs. 113Tyr-allele	1.35 [1.04, 1.75]	2.28 (0.02)	58.56	10 (<0.001)	83%	Random
His/His vs. Tyr/Tyr	1.65 [1.07, 2.54]	2.28 (0.02)	44.87	10 (<0.001)	78%	Random
His/His vs. Tyr/His+Tyr/Tyr	1.54 [1.21, 1.96]	3.51 (<0.001)	19.34	10 (0.04)	48%	Random
Tyr/Tyr vs. His/His+Tyr/His	0.73 [0.50, 1.07]	1.62 (0.11)	58.06	10 (<0.001)	83%	Random
**Ethnic subgroups**						
**Chinese (1070 cases, 1838 controls)**						
113His-allele vs. 113Tyr-allele	1.62 [1.15, 2.28]	2.76 (0.006)	28.65	5 (<0.001)	83%	Random
His/His vs. Tyr/Tyr	1.79 [1.08, 2.97]	2.25 (0.02)	19.27	5 (0.002)	74%	Random
His/His vs. Tyr/His+Tyr/Tyr	1.52 [1.26, 1.83]	4.40 (<0.001)	2.48	5 (0.78)	0%	Fixed
Tyr/Tyr vs. His/His+Tyr/His	0.63 [0.35, 1.13]	1.54 (0.12)	32.43	5 (<0.001)	85%	Random
**Others (UK, Sudan, Italy, Gambia, and India) (577 cases, 1762 controls)**						
113His-allele vs. 113Tyr-allele	1.07 [0.76, 1.51]	0.39 (0.70)	16.69	4 (0.002)	76%	Random
His/His vs. Tyr/Tyr	1.29 [0.61, 2.71]	0.67 (0.50)	18.11	4 (0.001)	78%	Random
His/His vs. Tyr/His+Tyr/Tyr	1.31 [0.76, 2.26]	0.97 (0.33)	11.11	4 (0.03)	64%	Random
Tyr/Tyr vs. His/His+Tyr/His	0.92 [0.59, 1.42]	0.39 (0.70)	15.13	4 (0.004)	74%	Random

For the heterogeneity of His139Arg mEH, statistically significant heterogeneity was observed for allelic contrast (*P* = 0.003, *I* ^2^ = 72%) and the dominant genetic model comparison (*P*<0.001, *I* ^2^ = 76%). However, no heterogeneity was found between studies for the homozygote comparison and recessive genetic model comparison (*I* ^2^ = 0%) (Table S1).

### Quantitative Data Synthesis

Table 3 shows the summary ORs for the Tyr113His mEH polymorphism and HCC risk on the basis of 1,696 HCC cases and 3,600 controls. We observed an association between Tyr113His mEH genotype and HCC risk in the total population based on all 11 studies. Given the ethnic differences in the allele frequency of this sequence variant, we evaluated the effect of the Tyr113His mEH polymorphism separately in Chinese and in other populations. Given that not only some of the healthy population in the control arm had liver disease, but also most studies did not report detailed data on the percentages of their subjects with these background conditions, we did not compute overall ORs stratified by source of control.

### Total Population

**Figure 2:**
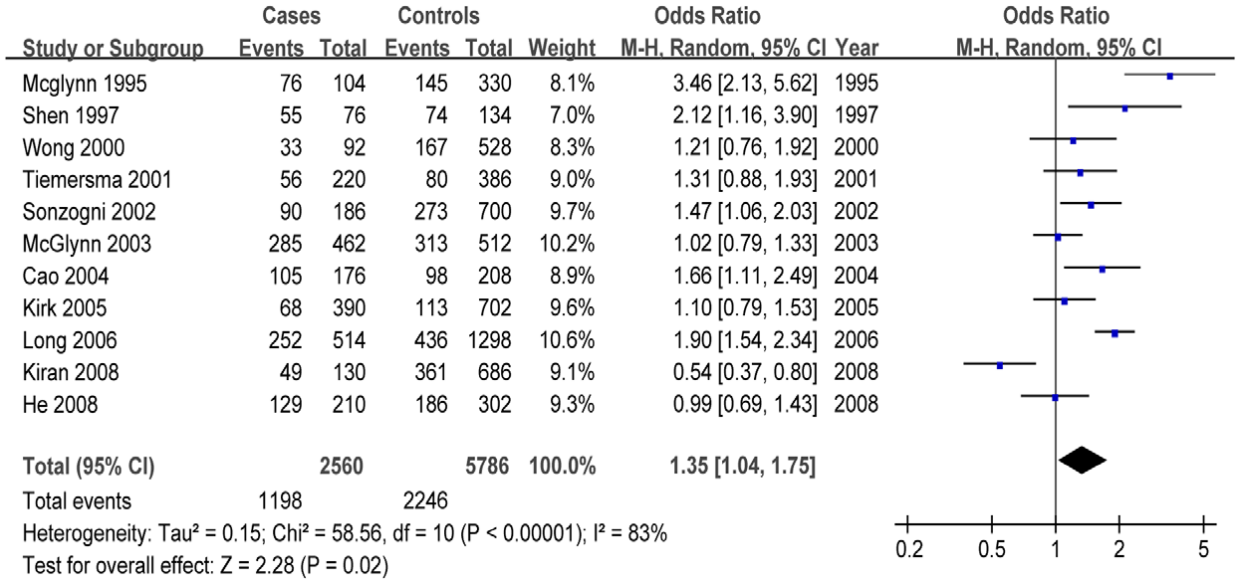
Forest plots describing the association of mEH polymorphism Tyr113His with hepatocellular carcinoma (113His- vs. 113Tyr-).

Calculation of overall OR in the total population using the random-effect model showed that the 113His- allele was strongly associated with increased risk of HCC based on allelic contrast (OR = 1.35, 95% CI = 1.04–1.75, *P = *0.02; *I* ^2^ = 83%) (Fig. 2), homozygote comparison (OR = 1.65, 95% CI = 1.07–2.54, *P = *0.02; *I* ^2^ = 78%) and the recessive genetic model (OR = 1.54, 95% CI = 1.21–1.96, *P*<0.001; *I* ^2^ = 48%). Association of the mEH with HCC risk was not observed in the total population using the dominant genetic model (OR = 0.73, 95% CI = 0.50–1.07, *P = *0.11; *I* ^2^ = 83%).

For the His139Arg mEH polymorphism and HCC risk, no statistically significant effect was observed based on allelic contrast (OR = 0.83, 95% CI = 0.59–1.18, *P = *0.30; *I* ^2^ = 72%), homozygote comparison (OR = 1.09, 95% CI = 0.67–1.78, *P = *0.74; *I* ^2^ = 0%), dominant genetic model comparison (OR = 1.40, 95% CI = 0.88–2.23, *P = *0.15; *I* ^2^ = 76%) or recessive genetic model comparison (OR = 1.08, 95% CI = 0.68–1.70, *P = *0.75; *I* ^2^ = 0%). Because the populations in the six studies reporting the His139Arg polymorphism were from non-overlapping ethnicities, we did not conduct subgroup analysis by ethnicity.

### Ethnicity

#### Chinese population

After stratification for ethnicity, we observed that in the Chinese population, the 113His- allele, homozygote variant and recessive genetic model were significantly associated with increased risk of HCC (113His- allele, OR = 1.62, 95% CI = 1.15–2.28, *P = *0.006; homozygote, OR = 1.79, 95% CI = 1.08–2.97, *P = *0.002; recessive model, OR = 1.52, 95% CI = 1.26–1.83, *P*<0.001). However, this association was not observed in the dominant genetic model (OR = 0.63, 95% CI = 0.35–1.13, *P = *0.12).

#### Mixed population

Analysis of the mixed population in five studies [12]–[16], [19] revealed that the 113His- allele, homozygote variant and recessive genetic model were not significantly associated with increased risk of HCC using a random-effect model (113His- allele, OR = 1.07, 95% CI = 0.76–1.51, *P = *0.70; homozygote, OR = 1.29, 95% CI = 0.61–2.71, *P = *0.50; recessive model, OR = 1.31, 95% CI = 0.76–2.26, *P = *0.33). Similarly, the dominant Tyr113His genotype was not associated with HCC risk.

### Sensitivity Analysis

In the allele contrast, the result was altered after excluding the study by Mcglynn and coworkers [10], with OR of 1.24 (95% CI 0.98 to 1.58, *P* = 0.08). In the recessive genetic model contrast, the results were not altered after excluding studies one by one. However, in the dominant genetic model contrast, the result was statistically significant after excluding the study by Kiran *et al*. [19], with OR of 0.66 (95% CI 0.46 to 0.94, *P* = 0.02). Therefore, the results should be interpreted with caution. For the His139Arg mEH polymorphism and HCC risk, the results were not altered based on allelic contrast, dominant or recessive genetic model comparison.

### Small-study Bias

**Figure 3:**
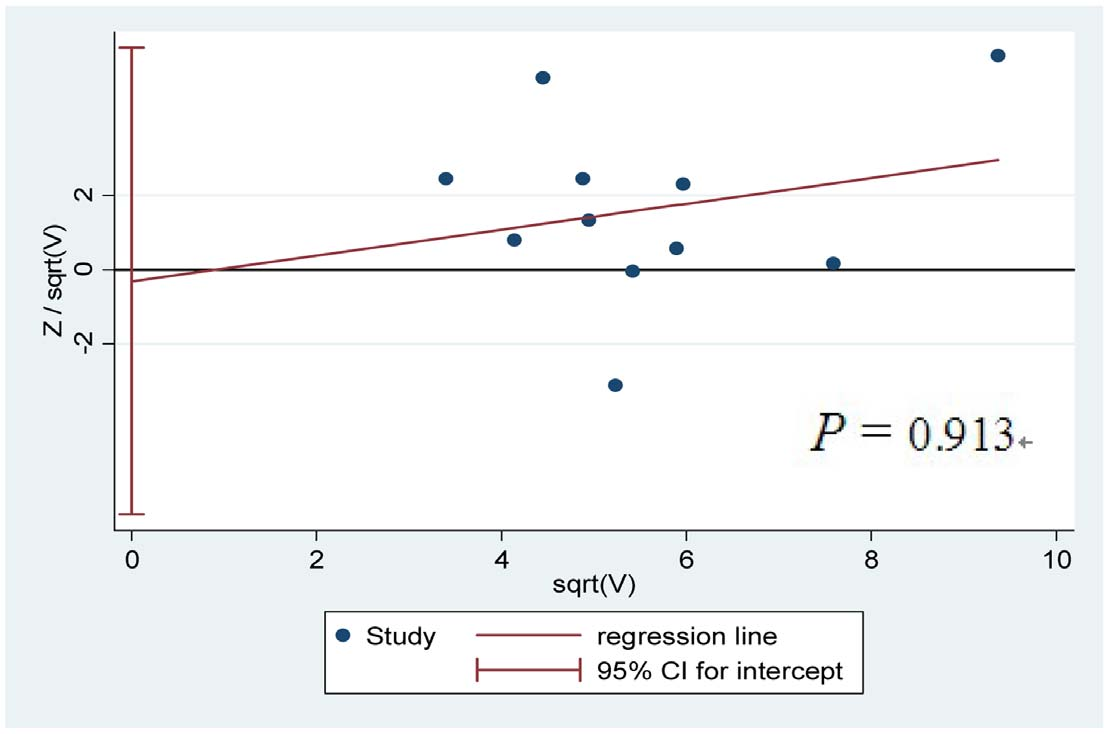
Small-study biases about the allele contrast (113His- vs. 113Tyr-) of mEH polymorphism with hepatocellular carcinoma.

Harbord’s modified test was prepared for the 11 studies [10]–[20] to assess the small-study bias for reported comparisons of mEH allele contrast and HCC. The P value is 0.913, suggesting the absence of small-study bias (Fig. 3).

## Discussion

HCC involves complex, multistep and heterogeneous malignant tumorigenesis. The pathogenesis of HCC involves host and environmental factors, as well as modulation of molecular signaling pathways implicated in malignant transformation of hepatocytes and tumor progression [25]. Polymorphism in the mEH gene has been associated with risk of various malignancies [26]–[28], including HCC [14]. Two relatively common genetic polymorphisms occur in the mEH gene: Tyr113His in exon 3 and His139Arg in exon 4. The C to T transition in the Tyr113His polymorphism eliminates an EcoRV restriction site, while an A to G transition in the His139Arg polymorphism creates an Rsa1 cleavage site [9]. The Tyr113His polymorphism decreases mEH activity by 40–50%, while the His139Arg polymorphism increases it by 25% [9].

Some studies reported an association between the Tyr113His polymorphism and HCC risk, while others found no such association. The most likely reason for the inconsistencies among these studies is that they are single case-control studies with small sample sizes. To help resolve these conflicting results using a larger sample size, we conducted meta-analysis of published studies. Our results for the total population suggest an increased HCC risk for subjects carrying the Tyr113His mEH genotype. Our approach also allowed us to look for potential ethnic differences in the association. Analysis of ethnic subgroups showed that in the Chinese population, the 113His- allele was strongly associated with increased risk of HCC based on allelic contrast, homozygote comparison and the recessive genetic model. However, no association was observed for other populations (UK, Sudan, Italy, Gambia, and India). Six studies [12]–[16], [19] reported the association of His139Arg mEH polymorphism and HCC risk. The results of the present meta-analysis revealed that the 139Arg- allele is not associated with increased or decreased risk of HCC.

The clinical significance of mEH polymorphisms extends beyond HCC. Polymorphisms in this gene have been reported to be susceptibility factors for several human diseases, such as type 2 diabetes mellitus [29], alcohol dependence [30], and chronic obstructive pulmonary disease [31]. In fact, two earlier meta-analyses [32]–[33] suggested a potential protective effect of the Tyr113His polymorphism and a potential harmful effect of the His139Arg polymorphism on lung cancer. While some studies have reported that mEH polymorphisms do not affect susceptibility to esophageal carcinoma [34] or colorectal cancer [35]–[36], other studies [27], [37] have shown that the mEH 113His allele is associated with increased risk of ovarian cancer.

Several studies in our meta-analysis indicate that mEH polymorphisms can interact with environmental factors to module HCC risk (Table 4) [10], [16], [18], [20]. Two such synergistic gene-environment interactions are that smoking populations with mEH polymorphism are at increased risk of HCC [16], [20], while a synergistic effect of smoking, drinking, and mEH polymorphism was observed in HBV-infected individuals [20]. Moreover, the association between mEH polymorphism and HCC depended on HBV status, suggesting a synergistic increase in risk of HCC when HBV infection and susceptible genotype occur together [10], [18].

**Table 4:** Studies reporting gene-gene or gene-environment interactions.

Study	Percent of liver disease cases with the indicated infection	Gene-gene or gene-environment interactions reported
Mcglynn 1995 (10)	77% HBV	The relationship of *mEH* polymorphism to HCC depended on HBV status, suggesting a synergistic effect.
Tiemersma 2001 (13)	41% HBV, 12% HCV	Individuals with *GSTM1* or *GSTT1* non-null genotypes in combination with the *113HH* and *139HH mEH* genotypes were at increased risk of HCC.
Cao 2004 (16)	NR	Smokers with a *mEH* polymorphism were at increased risk of HCC.
Kirk 2005 (17)	61% HBV, 19% HCV	Individuals who had all three suspected AFB-related high-risk genotypes (*GSTM1*-null, *mEH-HY/HH*, and *XRCC1-AG/GG*) had, 15-fold higher risk of HCC.
Long 2006 (18)	84% HBV	Individuals with more high-risk genotypes (*GSTM1*-null, *mEH-HY/HH*, *XRCC1-AG/GG*) faced a greater risk of HCC than those individuals without any high-risk genotype, especially among individuals with higher AFB exposure.
Kiran 2008 (19)	65% HBV, 35% HCV	Individuals with *GSTM1* or *GSTT1* non-null genotypes in combination with the *mEH 113HH* and *139HH* genotypes were at increased risk of HCC.
He 2008 (20)	74% HBV	Smokers with a *mEH* polymorphism were at increased risk of HCC; a synergistic effect of smoking, drinking, and *mEH* polymorphism was observed in HBV-infected individuals.

Note: HBV, hepatitis B virus; HCV, hepatitis C virus; HCC, hepatocellular carcinoma; mEH, microsomal epoxide hydrolase; AFB, aflatoxin B1; GST, glutathione-*S*-transferase; NR, not reported.

Our meta-analysis also revealed gene-gene interactions between the mEH polymorphisms and genotypes associated with elevated risk of AFB toxicity. Individuals with a greater number of suspected AFB-related high-risk genotypes (GSTM1-null, mEH-YH/HH, XRCC1-AG/GG) faced a greater risk of HCC than those without any high-risk genotypes, especially among individuals with higher AFB exposure [13], [17]–[19]. These results demonstrate that the etiology of HCC is complex and involves host and environmental factors that may interact synergistically.

The 113His codon variant is relatively common in the mEH gene. However, the allele frequency differs significantly among ethnicities. Kiyohara et al. [38] reported that frequency of the mEH 113His allele is greatest in the Asian population (51.2%), intermediate in Caucasians (30–40%), and lowest in African Americans (19.3%). Analyzing populations different from those of Kiyohara et al., Pinarbasi and coworkers [28] also observed that the rate of homozygous His113His is greatest in the Asian population (18–42%) and intermediate in the European population (about 10%). These findings are consistent with our meta-analysis. In our analysis, the Chinese population had the highest frequency of His113His: 35–56% among HCC cases and 26–44% among healthy individuals and individuals with other liver diseases. The frequencies of His113His in populations from the UK, Sudan, Italy, and India were 11–28% among HCC cases and 7–29% among healthy individuals and individuals with other liver diseases. The frequency in the Gambian population was lowest at 5% (Table 1).

Our data revealed that His113His mEH is a risk factor for hepatocarcinogenesis, but the complete picture is more complex. Both China and Gambia have among the highest incidences of HCC in the world, as well as a high prevalence of HBV infection and dietary exposure to AFB, which are the two main risk factors for HCC [39]–[41]. Nevertheless, Chinese had the highest frequency of His113His in our meta-analysis, while Gambians had the lowest. This discrepancy indicates that hepatocarcinogenesis has a complex, multifactorial etiology. It may also reflect the possibility that mEH mitigates the carcinogenic effects of AFB [42]. Polymorphisms in numerous other genes, such as those encoding epidermal growth factor [43] and tumor necrosis factor-alpha [44] are also associated with the risk of HCC. It may be that any single nucleotide polymorphism such as His113His is insufficient on its own to cause HCC, though it does increase the risk of the disease.

Of the total number of 5,296 subjects considered in the meta-analysis, at least 2,000 (37.8%) had one or more of the following: alcoholic liver disease, HBV or HCV infection, and cirrhosis. Since the studies in our meta-analysis often did not report detailed statistics on the proportion of HCC or control subjects with these background conditions, we could not perform subgroup analysis to separate the contribution of mEH polymorphism from that of possible confounders like HBV or HCV infection. Some other limitations of this meta-analysis also should be considered when interpreting the results. Bias may result from our exclusion of unpublished data, as well as papers published in languages other than English and Chinese [45]. Second, the results may be affected by additional confounding factors, such as tumor status, age or gender, but most studies either did not report these baseline data or aggregated them in different ways, making it impossible to include them in the meta-analysis. Third, there was significant heterogeneity among the studies. Fourth, five studies were based on non-overlapping countries and ethnicities and relied on quite small samples. Fifth, the result in the allelic contrast was altered after excluding one study. Therefore, the result should be explained with caution. Moreover, the distribution of genotypes among controls did not show HWE in several studies.

In conclusion, this meta-analysis suggests that the His113His mEH polymorphism may be associated with increased risk of HCC, especially in the Chinese population. Moreover, the mEH gene is involved in synergistic gene-gene and gene-environment interactions. These results suggest that sequence variation in exon 3 of the mEH gene may play an important role in the occurrence of HCC. In contrast, no association was observed between mEH gene variation in exon 4 and the risk of HCC. However, since this meta-analysis included few studies from non-Asian populations, large, well-designed studies in Caucasian and African populations are warranted to re-evaluate these associations.

This meta-analysis is guided by the PRISMA statement (Checklist S1).

## Supporting Information

Table S1 Overall and stratified meta-analyses of the association between mEH polymorphism His139Arg and risk of hepatocellular carcinoma.
(DOC)

## References

[1] Jemal A, Bray F (2011) Center MM, Ferlay J, Ward E, et al (2011) Global cancer statistics. CA Cancer J Clin 61: 69–90. 21296855 10.3322/caac.20107

[2] Nordenstedt H, White DL, El-Serag HB (2010) The changing pattern of epidemiology in hepatocellular carcinoma. Dig Liver Dis 42 Suppl 3S206–214. 20547305 10.1016/S1590-8658(10)60507-5 PMC3392755

[3] Kirk GD, Lesi OA, Mendy M, Akano AO, Sam O, et al (2004) The Gambia Liver Cancer Study: Infection with hepatitis B and C and the risk of hepatocellular carcinoma in West Africa. Hepatology 39: 211–219. 14752840 10.1002/hep.20027

[4] Velazquez RF, Rodriguez M, Navascues CA, Linares A, Pérez R, et al (2003) Prospective analysis of risk factors for hepatocellular carcinoma in patients with liver cirrhosis. Hepatology 37: 520–527. 12601348 10.1053/jhep.2003.50093

[5] Coleman WB (2003) Mechanisms of human hepatocarcinogenesis. Curr Mol Med 3: 573–588. 14527088 10.2174/1566524033479546

[6] Newman JW, Morisseau C, Hammock BD (2005) Epoxide hydrolases: their roles and interactions with lipid metabolism. Prog Lipid Res 44: 1–51. 15748653 10.1016/j.plipres.2004.10.001

[7] Fretland AJ, Omiecinski CJ (2000) Epoxide hydrolases: biochemistry and molecular biology. Chem Biol Interact 129: 41–59. 11154734 10.1016/s0009-2797(00)00197-6

[8] Shou M, Gonzalez FJ, Gelboin HV (1996) Stereoselective epoxidation and hydration at the K-region of polycyclic aromatic hydrocarbons by cDNA-expressed cytochromes P450 1A1, 1A2, and epoxide hydrolase. Biochemistry 35: 15807–813. 8961944 10.1021/bi962042z

[9] Hassett C, Aicher L, Sidhu JS, Omiecinski CJ (1994) Human microsomal epoxide hydrolase: genetic polymorphism and functional expression in vitro of amino acid variants. Hum Mol Genet 3: 421–428. 7516776 10.1093/hmg/3.3.421 PMC4868095

[10] McGlynn KA, Rosvold EA, Lustbader ED, Hu Y, Clapper ML, et al (1995) Susceptibility to hepatocellular carcinoma is associated with genetic variation in the enzymatic detoxification of aflatoxin B1. Proc Natl Acad Sci U S A 92: 2384–2387. 7892276 10.1073/pnas.92.6.2384 PMC42488

[11] Shen F, Hu Y (1997) Association between the gene polymorphism of epoxide hydrolase and the susceptibility to primary hepatocellular carcinoma. Chin J Med Genet 14: 96–98.

[12] Wong NA, Rae F, Bathgate A, Smith CA, Harrison DJ (2000) Polymorphisms of the gene for microsomal epoxide hydrolase and susceptibility to alcoholic liver disease and hepatocellular carcinoma in a Caucasian population. Toxicol Lett 115: 17–22. 10817627 10.1016/s0378-4274(00)00166-1

[13] Tiemersma EW, Omer RE, Bunschoten A, van't Veer P, Kok FJ, et al (2001) Role of genetic polymorphism of glutathione-S-transferase T1 and microsomal epoxide hydrolase in aflatoxin-associated hepatocellular carcinoma. Cancer Epidemiol Biomarkers Prev 10: 785–791. 11440964

[14] Sonzogni L, Silvestri L, De Silvestri A, Gritti C, Foti L, et al (2002) Polymorphisms of microsomal epoxide hydrolase gene and severity of HCV-related liver disease. Hepatology 36: 195–201. 12085365 10.1053/jhep.2002.33898

[15] McGlynn KA, Hunter K, LeVoyer T, Roush J, Wise P, et al (2003) Susceptibility to aflatoxin B1-related primary hepatocellular carcinoma in mice and humans. Cancer Res 63: 4594–4601. 12907637

[16] Cao YY, Bian JC, Jiang F, Zhang ZM, Wang QM, et al (2004) Association between the genetic polymorphism of epoxide hydrolase and the genetic susceptibility to primary liver cancer. Fudan University Journal of Medical Sciences 31: 363–367.

[17] Kirk GD, Turner PC, Gong Y, Lesi OA, Mendy M, et al (2005) Hepatocellular carcinoma and polymorphisms in carcinogen-metabolizing and DNA repair enzymes in a population with aflatoxin exposure and hepatitis B virus endemicity. Cancer Epidemiol Biomarkers Prev 14: 373–379. 15734960 10.1158/1055-9965.EPI-04-0161

[18] Long XD, Ma Y, Wei YP, Deng ZL (2006) The polymorphisms of GSTM1, GSTT1, HYL1*2, and XRCC1, and aflatoxin B1-related hepatocellular carcinoma in Guangxi population, China. Hepatol Res 36: 48–55. 16884947 10.1016/j.hepres.2006.06.004

[19] Kiran M, Chawla YK, Kaur J (2008) Glutathione-S-transferase and microsomal epoxide hydrolase polymorphism and viral-related hepatocellular carcinoma risk in India. DNA Cell Biol 27: 687–694. 18816171 10.1089/dna.2008.0805

[20] He SJ, Gu YY, Lin WZ, Zeng XY, Liao ZH (2008) Polymorphism of microsomal epoxide hydrolase and susceptibility to primary hepatocellular carcinoma. Tumor 28: 125–128.

[21] Higgins JP, Thompson SG, Deeks JJ, Altman DG (2003) Measuring inconsistency in meta-analyses. BMJ 327: 557–560. 12958120 10.1136/bmj.327.7414.557 PMC192859

[22] Ioannidis JP, Trikalinos TA (2007) An exploratory test for an excess of significant findings. Clin Trials 4: 245–253. 17715249 10.1177/1740774507079441

[23] Kiran M, Chawla YK, Jain M, Kaur J (2009) Haplotypes of microsomal epoxide hydrolase and x-ray cross-complementing group 1 genes in Indian hepatocellular carcinoma patients. DNA Cell Biol 28: 573–577. 19754350 10.1089/dna.2009.0921

[24] Rahat B, Kiran M, Saxena R, Chawla YK, Sharma RR, et al (2012) Microsomal Epoxide Hydrolase Polymorphisms and Haplotypes as Determinants of Hepatitis B Virusand Hepatitis C Virus-related Liver Disease in Indian Population. J Clin Exp Hepatol 2: 104–111. 25755418 10.1016/S0973-6883(12)60097-8 PMC3940147

[25] Llovet JM, Bruix J (2008) Molecular targeted therapies in hepatocellular carcinoma. Hepatology 48: 1312–1327. 18821591 10.1002/hep.22506 PMC2597642

[26] Koutros S, Andreotti G, Berndt SI, Hughes BK, Lubin JH, et al (2011) Xenobiotic-metabolizing gene variants, pesticide use, and the risk of prostate cancer. Pharmacogenet Genomics 21: 615–623. 21716162 10.1097/FPC.0b013e3283493a57 PMC3172373

[27] Goode EL, White KL, Vierkant RA, Phelan CM, Cunningham JM, et al (2011) Xenobiotic-Metabolizing gene polymorphisms and ovarian cancer risk. Mol Carcinog 50: 397–402. 21480392 10.1002/mc.20714 PMC3115705

[28] Pinarbasi H, Silig Y, Pinarbasi E (2010) Microsomal epoxide hydrolase polymorphisms. Mol Med Report 3: 723–727. 10.3892/mmr_00000324 21472306

[29] Ghattas MH, Amer MA (2012) Possible role of microsomal epoxide hydrolase gene polymorphism as a risk factor for developing insulin resistance and type 2 diabetes mellitus. Endocrine [Epub ahead of print]. 10.1007/s12020-012-9656-5 22555758

[30] Bhaskar LV, Thangaraj K, Patel M, Shah AM, Gopal K, et al.. (2012) EPHX1 Gene Polymorphisms in Alcohol Dependence and their Distribution among the Indian Populations. Am J Drug Alcohol Abuse [Epub ahead of print]. 10.3109/00952990.2011.643991 22257321

[31] Chen CZ, Wang RH, Lee CH, Lin CC, Chang HY, et al (2011) Polymorphism of microsomal epoxide hydrolase is associated with chronic obstructive pulmonary disease and bronchodilator response. J Formos Med Assoc 110: 685–689. 22118311 10.1016/j.jfma.2011.09.003

[32] Lee WJ, Brennan P, Boffetta P, London SJ, Benhamou S, et al (2002) Microsomal epoxide hydrolase polymorphisms and lung cancer risk: a quantitative review. Biomarkers 7: 230–241. 12141066 10.1080/13547500210121882

[33] Li X, Hu Z, Qu X, Zhu J, Li L, et al (2011) Putative EPHX1 enzyme activity is related with risk of lung and upper aerodigestive tract cancers: a comprehensive meta-analysis. PLoS One 6: e14749. 21445251 10.1371/journal.pone.0014749 PMC3060809

[34] Dura P, Bregitha CV, Te Morsche RH, Roelofs HM, Kristinsson JO, et al (2012) EPHX1 polymorphisms do not modify esophageal carcinoma susceptibility in Dutch Caucasians. Oncol Rep 27: 1710–1716. 22447130 10.3892/or.2012.1734

[35] Nisa H, Budhathoki S, Morita M, Toyomura K, Nagano J, et al.. (2012) Microsomal epoxide hydrolase polymorphisms, cigarette smoking, and risk of colorectal cancer: The Fukuoka Colorectal Cancer Study. Mol Carcinog [Epub ahead of print]. 10.1002/mc.21897 22415791

[36] Zhao ZQ, Guan QK, Yang FY, Zhao P, Zhou B, et al (2012) System review and metaanalysis of the relationships between five metabolic gene polymorphisms and colorectal adenoma risk. Tumour Biol 33(2): 523–535. 22161138 10.1007/s13277-011-0287-x

[37] Baxter SW, Choong DY, Campbell IG (2002) Microsomal epoxide hydrolase polymorphism and susceptibility to ovarian cancer. Cancer Lett 177: 75–81. 11809533 10.1016/s0304-3835(01)00782-0

[38] Kiyohara C, Yoshimasu K, Takayama K, Nakanishi Y (2006) EPHX1 polymorphisms and the risk of lung cancer: a HuGE review. Epidemiology 17: 89–99. 16357600 10.1097/01.ede.0000187627.70026.23

[39] Wild CP, Jiang YZ, Allen SJ, Jansen LA, Hall AJ, et al (1990) Aflatoxin-albumin adducts in human sera from different regions of the world. Carcinogenesis 11: 2271–2274. 2265478 10.1093/carcin/11.12.2271

[40] Wild CP, Hudson GJ, Sabbioni G, Chapot B, Hall AJ, et al (1992) Dietary intake of aflatoxins and the level of albumin-bound aflatoxin in peripheral blood in The Gambia, West Africa. Cancer Epidemiol Biomarkers Prev 1: 229–234. 1339083

[41] Vall Mayans M, Hall AJ, Inskip HM, Chotard J, Lindsay SW, et al (1990) Risk factors for transmission of hepatitis B virus to Gambian children. Lancet 336: 1107–1109. 1977989 10.1016/0140-6736(90)92580-b

[42] Kelly EJ, Erickson KE, Sengstag C, Eaton DL (2002) Expression of human microsomal epoxide hydrolase in Saccharomyces cerevisiae reveals a functional role in aflatoxin B1 detoxification. Toxicol Sci 65: 35–42. 11752683 10.1093/toxsci/65.1.35

[43] Zhong JH, You XM, Gong WF, Ma L, Zhang Y, et al (2012) Epidermal growth factor gene polymorphism and risk of hepatocellular carcinoma: a meta-analysis. PLoS One 7: e32159. 22403631 10.1371/journal.pone.0032159 PMC3293888

[44] Qin H, Liu B, Shi T, Liu Y, Sun Y, et al (2010) Tumour necrosis factor-alpha polymorphisms and hepatocellular carcinoma: a meta-analysis. J Int Med Res 38: 760–768. 20819413 10.1177/147323001003800304

[45] Pan Z, Trikalinos TA, Kavvoura FK, Lau J, Ioannidis JP (2005) Local literature bias in genetic epidemiology: an empirical evaluation of the Chinese literature. PLoS Med 2: e334. 16285839 10.1371/journal.pmed.0020334 PMC1285066

