# Supplementary material for: Meta-Analysis of Microsomal Epoxide Hydrolase Gene Polymorphism and Risk of Hepatocellular Carcinoma
Source: PLoS One. 2013 Feb 25;8(2):e57064. doi: 10.1371/journal.pone.0057064 (PMC3581564; doi:10.1371/journal.pone.0057064)
Supplement: Table S1 — Overall and stratified meta-analyses of the association between mEH polymorphism His139Arg and risk of hepatocellular carcinoma. (DOC) [file pone.0057064.s001.doc]

**Table S1.** Overall and stratified meta-analyses of the association between mEH polymorphism His139Arg and risk of hepatocellular carcinoma.

| **Genotype comparison** | **OR [95% CI]** | **Z (P value)** | **Heterogeneity of study design** | | | **Analysis model** |
| --- | --- | --- | --- | --- | --- | --- |
|  |  |  | **χ^2^** | **df (P value)** | **I^2^** |  |
| **626 cases, 1561 controls** | | | | | | |
| 139Arg-allele vs. 139His-allele | 0.83 [0.59, 1.18] | 1.04 (0.30) | 17.82 | 5 (0.003) | 72% | Random |
| Risk vs. Non-risk | 1.09 [0.67, 1.78] | 0.34 (0.74) | 4.96 | 5 (0.42) | 0% | Fixed |
| Risk vs. Heterozygous + Non-risk | 1.08 [0.68, 1.70] | 0.32 (0.75) | 3.54 | 5 (0.62) | 0% | Fixed |
| Non-risk vs. Risk + Heterozygous | 1.40 [0.88, 2.23] | 1.42 (0.15) | 20.71 | 5 (＜0.001) | 76% | Random |
